# Cholesterol-dependent Conformational Plasticity in GPCR Dimers

**DOI:** 10.1038/srep31858

**Published:** 2016-08-18

**Authors:** Xavier Prasanna, Durba Sengupta, Amitabha Chattopadhyay

**Affiliations:** 1 CSIR-National Chemical Laboratory, Dr. Homi Bhabha Road, Pune 411 008, India; 2 CSIR-Centre for Cellular and Molecular Biology, Uppal Road, Hyderabad 500 007, India

## Abstract

The organization and function of the serotonin_1A_ receptor, an important member of the GPCR family, have been shown to be cholesterol-dependent, although the molecular mechanism is not clear. We performed a comprehensive structural and dynamic analysis of dimerization of the serotonin_1A_ receptor by coarse-grain molecular dynamics simulations totaling 3.6 ms to explore the molecular details of its cholesterol-dependent association. A major finding is that the plasticity and flexibility of the receptor dimers increase with increased cholesterol concentration. In particular, a dimer interface formed by transmembrane helices I-I was found to be sensitive to cholesterol. The modulation of dimer interface appears to arise from a combination of direct cholesterol occupancy and indirect membrane effects. Interestingly, the presence of cholesterol at the dimer interface is correlated with increased dimer plasticity and flexibility. These results represent an important step in characterizing the molecular interactions in GPCR organization with potential relevance to therapeutic interventions.

G protein-coupled receptors (GPCRs) are lipid-dependent membrane receptors^1^,^2^,^3^,^4^ that constitute the largest family of current therapeutic targets^5^,^6^. The major function of GPCRs is information transfer across the cellular plasma membrane upon activation by a ligand, and the subsequent regulation of a large number of physiological processes. Molecular details on GPCR conformational states have begun to emerge as a result of recent success in structure determination of several GPCRs^7^,^8^,^9^. In addition, recent advances in computational and NMR approaches have helped uncover molecular mechanisms of receptor activation^10^,^11^,^12^,^13^,^14^,^15^,^16^. On the other hand, the molecular organization of GPCRs, especially in the context of their physiological role, is less explored^17^,^18^. Recent studies have shown that the oligomerization of certain GPCRs is dynamic^19^,^20^ and constitutive^21^,^22^. Membrane lipids (particularly cholesterol)^22^,^23^,^24^,^25^ and the cytoskeletal network^22^,^25^ have been implicated in the modulation of GPCR function and oligomerization. Receptor oligomerization has been suggested to increase the cross-talk between receptors^26^ and potential downstream signaling capabilities of GPCRs, thereby providing a framework for efficient and controlled signal transduction^17^. Receptor oligomerization assumes greater significance for better therapeutic strategies and recent exploratory studies have confirmed the increased specificity of multivalent drugs^27^, as well as ligand sensitivity of the various dimer interfaces^28^. In this overall context, GPCR oligomerization is an emerging paradigm, and needs to be explored in detail to improve our understanding of GPCR function in health and disease.

The serotonin_1A_ receptor is an important neurotransmitter receptor that is implicated in various cognitive, behavioral, and developmental functions^29^,^30^. The agonists and antagonists of the serotonin_1A_ receptor represent major classes of molecules with potential therapeutic applications in anxiety- or stress-related disorders^31^. As a result, the serotonin_1A_ receptor serves as an important drug target for neuropsychiatric disorders such as anxiety and depression as well as in neuronal developmental defects^32^. It is one of the first receptors for which cholesterol dependence of ligand binding and signaling function was demonstrated^reviewed in^ 33,34,35. Highly dynamic cholesterol interactions have been identified on the receptor surface using coarse-grain simulations^36^,^37^. The serotonin_1A_ receptor has been shown to oligomerize in a constitutive manner^22^ which is dependent on membrane cholesterol content^22^,^23^. However, the molecular interplay between membrane cholesterol and receptor oligomerization is still lacking.

In this work, we have used coarse-grain molecular dynamics simulations to analyze the dimerization of the serotonin_1A_ receptor in membranes of varying cholesterol content. A major finding from our results is the high conformational plasticity of the dimer with increasing cholesterol concentration. We postulate that increased cholesterol concentration at the interface between the two receptors, reminiscent of ‘nonannular’ sites^38^, is responsible for the increased dimer rotational flexibility and plasticity. These results help explain the molecular mechanism governing cholesterol-dependent receptor oligomerization. We believe these results provide an important first step toward the design of therapeutic strategies that could be exploited for tissue-specific and age-dependent interventions.

## Results

The dimerization of membrane-embedded serotonin_1A_ receptors was analyzed from a series of coarse-grain molecular dynamics simulations, totaling to 3.6 ms of simulation time. To investigate the dependence of receptor dimerization on membrane lipid composition, simulations were performed in POPC bilayers and POPC/cholesterol bilayers with increasing cholesterol concentration. A schematic representation of the receptor and a representative initial system are shown in Supplementary Fig. 1. Twenty independent simulations of 45 μs were performed for each membrane composition for the receptor dimer (Supplementary Table 1).

### Receptor association is dependent on cholesterol concentration

**Figure 1:**
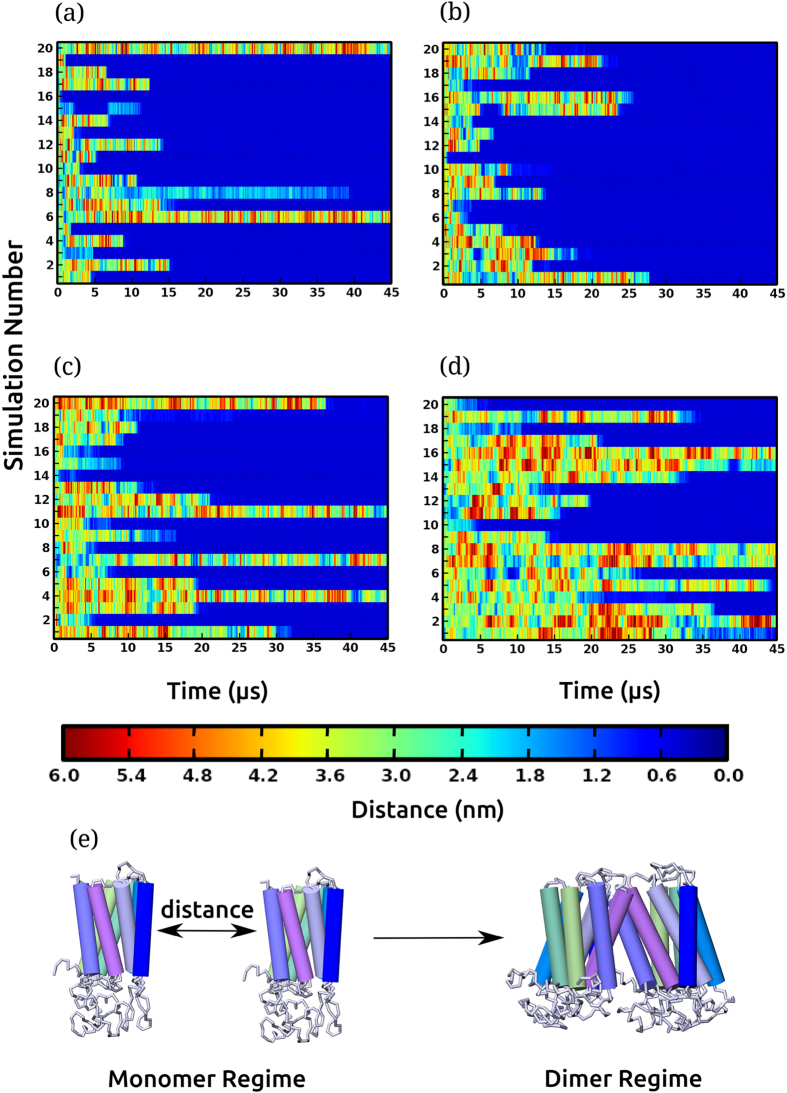
Serotonin_1A_ receptor dimerization. Schematic representations of the minimum distance between the transmembrane segments of the two receptors during the course of the simulation in (**a**) POPC bilayers and POPC/cholesterol bilayers with (**b**) 9 (**c**) 30 and (**d**) 50% cholesterol concentrations. The range of distances between the monomers is color coded and shown as a scale bar. The dimer regime is characterized by distances less than 0.5 nm, corresponding to the dark blue stretches in the plot. The monomer regime corresponds to the red, yellow, green and light blue regions in the plot. Each row in every panel represents an independent simulation (numbered along the ordinate), thereby corresponding to a total of 80 simulations of 45 μs each. (**e**) A schematic representation of the two receptors in the monomer and dimer regime. See Methods for other details.

During the course of simulation, the receptors diffused freely, with a μs time scale encounter frequency. A time-distance plot showing the minimum distance between the two receptors for each simulation is shown in Fig. 1. The monomeric regime when the two receptors diffuse independently is characterized by distances larger than 1 nm. The dimer regime corresponds to smaller distances (<0.5 nm closest approach), depicted by the dark blue stretches in Fig. 1. Receptor dimerization was observed in all membrane compositions. In most cases, several close associations between the two receptors were observed prior to dimer formation. The time taken to form a dimer was variable and ranged from 1 to 45 μs. The most favorable long-lived dimer species were stable in the time scale of the simulations, although transient dimer species were observed as well. Interestingly, the number of dimers observed in the simulations exhibited a dependence on cholesterol concentration. At higher cholesterol concentrations (30 and 50%), the number of dimer species observed was lower relative to what was observed in POPC bilayers and POPC/cholesterol bilayers with 9% cholesterol. For example, only 14 long-lived stable dimers (in a total of 20 simulations) were observed in POPC/cholesterol bilayers with 50% cholesterol. In addition, dimerization was less frequent at higher cholesterol concentrations, as evident from the length of the dark blue stretches in Fig. 1.

### Cholesterol increases plasticity of dimer conformers

**Figure 2:**
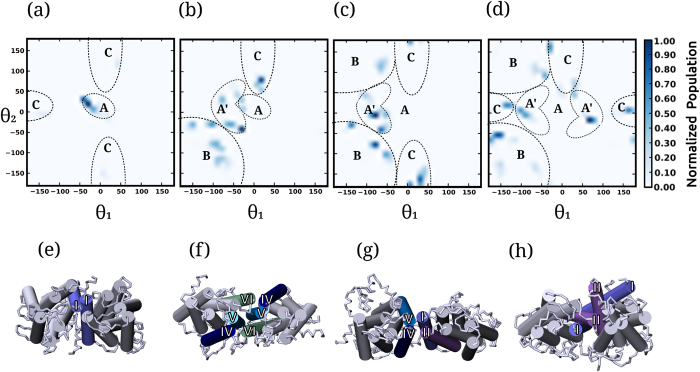
Relative orientations of the receptors in the dimer state. Normalized population of the relative orientations of the two receptors in the dimer regime defined by the angles θ_1_ and θ_2_ (see Methods for details). The populations were averaged over the dimer regime for simulations in (**a**) POPC bilayers and POPC/cholesterol bilayers with (**b**) 9 (**c**) 30 and (**d**) 50% cholesterol concentrations. The relative orientations can be broadly mapped to four conformations (A, B, C and A′) that are marked in panels (**a**–**d**). The four conformations correspond to two sites at the receptor: site 1 comprising of transmembrane helices I and II and site 2 comprising of transmembrane helices IV, V and VI. (**e**) Conformation A corresponds to a non-flexible homo-interface with only a single helix from site 1 (transmembrane helix I). (**f**) Conformation B corresponds to homo-interfaces at site 2. (**g**) Conformation C corresponds to hetero-interfaces comprising of transmembrane helices from sites 1 and 2. (**h**) Conformation A′ corresponds to a flexible interface at site 1.

To analyze the dimer conformations observed in the simulations, we calculated the relative orientations of the receptors in the dimer regime. The conformations were characterized by measuring the rotational angle of the two receptors relative to each other, defined by two arbitrary angles θ_1_ and θ_2_ for the two receptors (See Methods and Supplementary Fig. 2 for further details). Two-dimensional plots of the population densities of the relative orientations at each membrane composition are shown in Fig. 2. The most striking feature of the rotational orientations sampled is that only a few of the conformations were sampled in POPC bilayers and the conformational diversity appeared to increase with increase in cholesterol concentration. We broadly mapped the relative orientations to four conformations (A, B, C and A′). A visual inspection of these four conformations revealed that they correspond to mainly two sites at the receptor: site 1 comprising of transmembrane helices I and II (and occasionally VII) and site 2 comprising of transmembrane helices IV, V and VI. Schematic representations of the conformers (*i.e*., A, B, C and A′) are shown in Fig. 2(e–h).

In POPC bilayers, the most favorable conformer was A (see Fig. 2a,e) which corresponds to a symmetric homodimer (values of θ_1_ and θ_2_ are close to zero). The interface was observed to have low rotational flexibility and consists of only transmembrane helix I from site 1 of the receptor. Additional conformations (B and C) were sampled, but with reduced population. In POPC/cholesterol bilayers with 9% cholesterol (Fig. 2b), several dimer conformations were sampled. Conformer B (Fig. 2f) was observed to have a high population. A visual inspection revealed that conformer B correspond to several related conformers at site 2 (transmembrane helices IV, V and VI). These interfaces were observed to be very flexible and small rotations around each monomer resulted in variable transmembrane helices at the dimer interface. In addition, the population of conformer A decreased, but a related conformer A′ (Fig. 2h) was found. Conformer A′ corresponds to a more flexible interface than conformer A, but at the same site 1 of the receptor. At increased cholesterol concentrations (30 and 50%), several instances of conformer C (Fig. 2g) were sampled. Conformer C corresponds to heterointerfaces of helices from sites 1 and 2. Interestingly, at these higher concentrations, conformer A (prominently observed in POPC bilayers) was not observed at all. In these cases, the more flexible conformer A′ was observed.

Taken together, the results suggest that cholesterol modulates dimer conformations in a way so as to populate the conformer space with more plastic and flexible dimers. This is particularly evident in case of the less flexible conformer A that was not observed at higher cholesterol concentrations (Fig. 2c,d). A related flexible conformer A′ was observed at higher cholesterol concentrations, but not in the absence of cholesterol (Fig. 2a). In addition, more conformers were sampled at increased cholesterol concentration, highlighting the role of cholesterol in the increased flexibility and plasticity of the dimer conformations.

### Cholesterol modulates the receptor dimer interface

**Figure 3:**
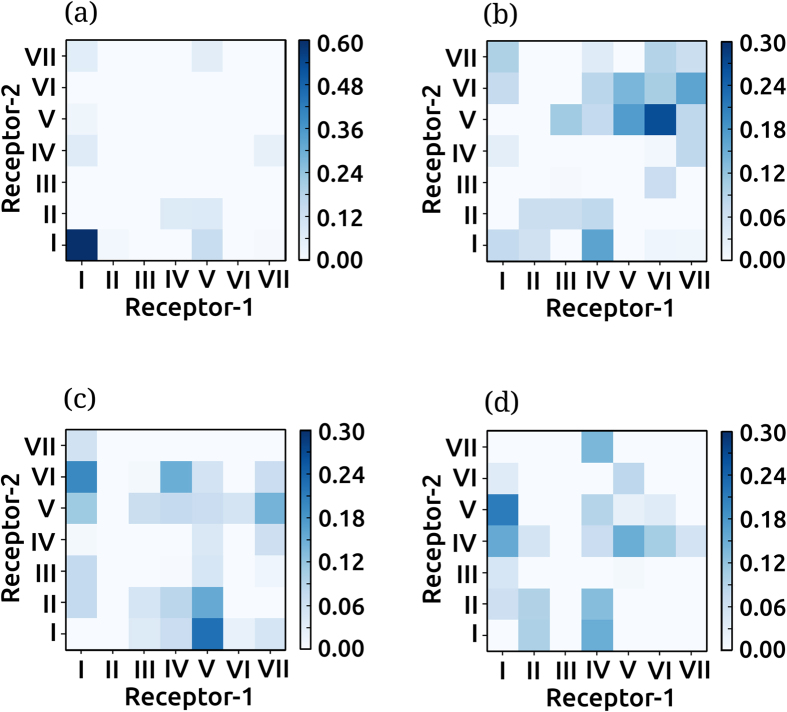
Characterizing the favorable dimer interfaces. Contact maps depicting the helix-helix interactions at the dimer interface in (**a**) POPC bilayers and POPC bilayers containing (**b**) 9 (**c**) 30 and (d) 50% cholesterol. The dimer interfaces were calculated from the long-lived stable dimers not exhibiting any subsequent dissociation in the time scale of the simulation. The values were calculated as an average over all simulations and normalized by the time of occurrence and simulation length. A cut-off distance of 0.5 nm was used to determine the contact residues. The color scale bar indicates the normalized population. For better clarity, the color scale bar for the dimer interfaces sampled in POPC bilayers is different from the POPC/cholesterol bilayers. See Methods for further details.

We further analyzed the dimer conformations and estimated the relative contributions of the individual transmembrane helices at the dimer interface. Only the long-lived stable dimer conformations that did not exhibit any subsequent dissociation were considered. Contact maps representing the transmembrane helix pairs at the dimer interfaces at different cholesterol concentrations are shown in Fig. 3. A distinct difference was observed in the contact maps with increasing cholesterol concentration in the membrane. The most striking feature that emerges is the predominant occurrence of the transmembrane helix I-I homodimer in POPC bilayers (see Fig. 3a). This interface was sampled less in POPC/cholesterol bilayers with 9% cholesterol (Fig. 3b) and was completely absent at higher cholesterol concentrations (Fig. 3c,d). Another prominent feature of the contact maps is the increased plasticity, *i.e*., presence of multiple favorable helix-helix contacts, in presence of cholesterol. The main features of the contact maps are in agreement with the rotational orientations sampled (Fig. 2), and additionally allow us to characterize the dimer interfaces at a molecular level.

### Unfavorable dimer interfaces dissociate at ns to μs time scale

A detailed analysis of the minimum distance between the receptors over the simulation period pointed to several dissociation events (Fig. 1). Several of the unfavorable dimers that dissociate had been stably bound at μs time scale, and are distinct from the multiple close-contacts formed prior to the formation of a long-lived dimer. One such example was observed in set 15 in POPC/cholesterol bilayers with 9% cholesterol (Fig. 1b). A dimer was observed for almost 3 μs between 4.8 and 7.8 μs, after which it dissociated. The two receptors freely diffused away, moving apart by as much as 6 nm (shown as red), and consequently dimerize again at 23 μs and remain associated until 45 μs. Another example of such a dissociation event was observed in set 6 in POPC/cholesterol bilayers with 50% cholesterol (see Fig. 1d). Four dimer association/dissociation events were observed, in which the dimer species was observed for at least 1 μs, followed by subsequent dissociation. The receptors were observed to diffuse away and finally re-associate after several μs. Taken together, ~124 dissociation events were observed, ranging from ns to μs time scale. The unstable dimer species was observed in all membrane compositions (see Fig. 1), but the number of dissociation events considerably increased with increasing cholesterol concentration (see Supplementary Table 2). In POPC/cholesterol bilayers with 50% cholesterol concentration, the number of dissociation events was the largest, despite a slower initial association (see Fig. 1d).

**Figure 4:**
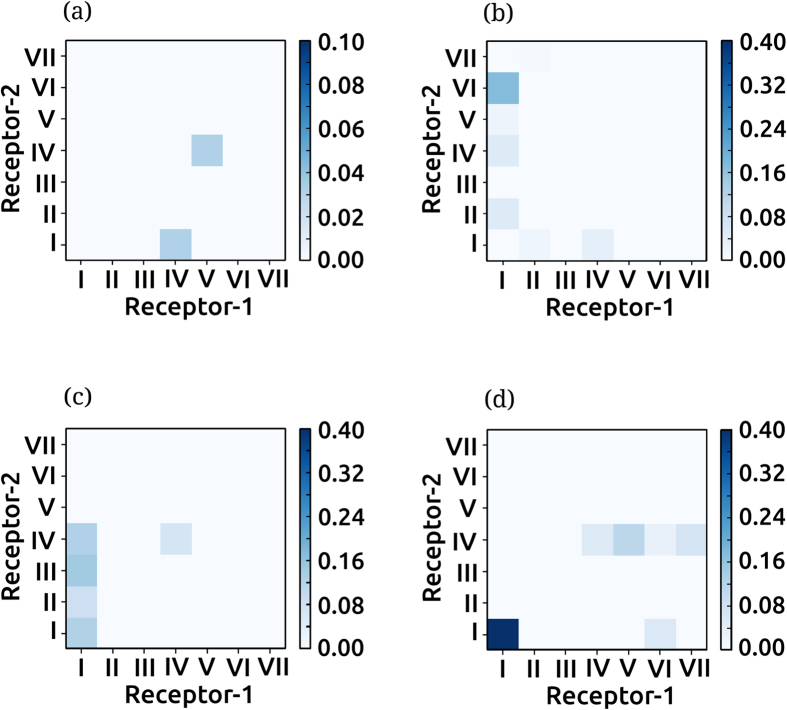
Characterizing the unfavorable dimer interfaces. Contact maps depicting the helix-helix interactions at the dimer interface for transiently associated dimers in (**a**) POPC bilayers and POPC bilayers containing (**b**) 9 (**c**) 30 and (**d**) 50% cholesterol. The dimer interfaces were calculated from the short-lived unstable dimers that subsequently dissociate during the simulation. The values were normalized to the transient association period and the maximum total number of transient association instances (see Supplementary Table 2). The color scale bar indicates the normalized population. For better clarity, the color scale bar for the dimer interfaces sampled in POPC bilayers is different from the POPC/cholesterol bilayers. See Methods for further details.

We characterized the nature of these transient interactions by analyzing the dimer conformers sampled during these short-lived unstable associations. Contact maps of the dimer conformers sampled during the unstable short-lived associations are shown in Fig. 4. In POPC bilayers, the dissociation events are low (Fig. 4a), and the transient dimer conformers consisted of transmembrane helices IV/I and V/IV. In POPC/cholesterol bilayers with 9% cholesterol concentration (see Fig. 4b), the transient dimer conformers consisted of transmembrane helix I in most cases, in combination with transmembrane helix II, IV and VI. In POPC/cholesterol bilayers with 30% cholesterol (Fig. 4c), the transient dimer conformers mostly included transmembrane helix I. In particular, the I-I homodimer was found to dissociate in several instances. In POPC/cholesterol bilayers with 50% cholesterol (Fig. 4d), the least favorable dimer conformer was the I-I homodimer.

Interestingly, the I-I homodimer, predominantly sampled in POPC bilayers (Fig. 3a), never dissociated within the μs time scales of the current simulations (Fig. 4a). In sharp contrast to this, in POPC/cholesterol bilayers with increased cholesterol concentrations (Fig. 3c,d), the I-I homodimer was not observed in the stable dimer regime and dissociated in case the initial contacts were formed (Fig. 4c,d). These results therefore indicate that the presence of cholesterol modulates both the initial approach between receptors and the relative stability of the favorable dimer conformers by fine-tuning the conformational energetics.

### Cholesterol modulates receptor dimerization through direct and indirect effects

To examine the molecular basis of the modulation of the dimer conformations by cholesterol, we analyzed the possible direct and indirect effects of cholesterol^39^. The direct effects arise from an interaction between the receptor and cholesterol and have been characterized by the occupancy of cholesterol around each residue of the receptor. Indirect effects arise from changes in the bilayer properties such as changes in bilayer thickness due to the presence of cholesterol.

#### Residue-wise cholesterol occupancy

**Figure 5:**
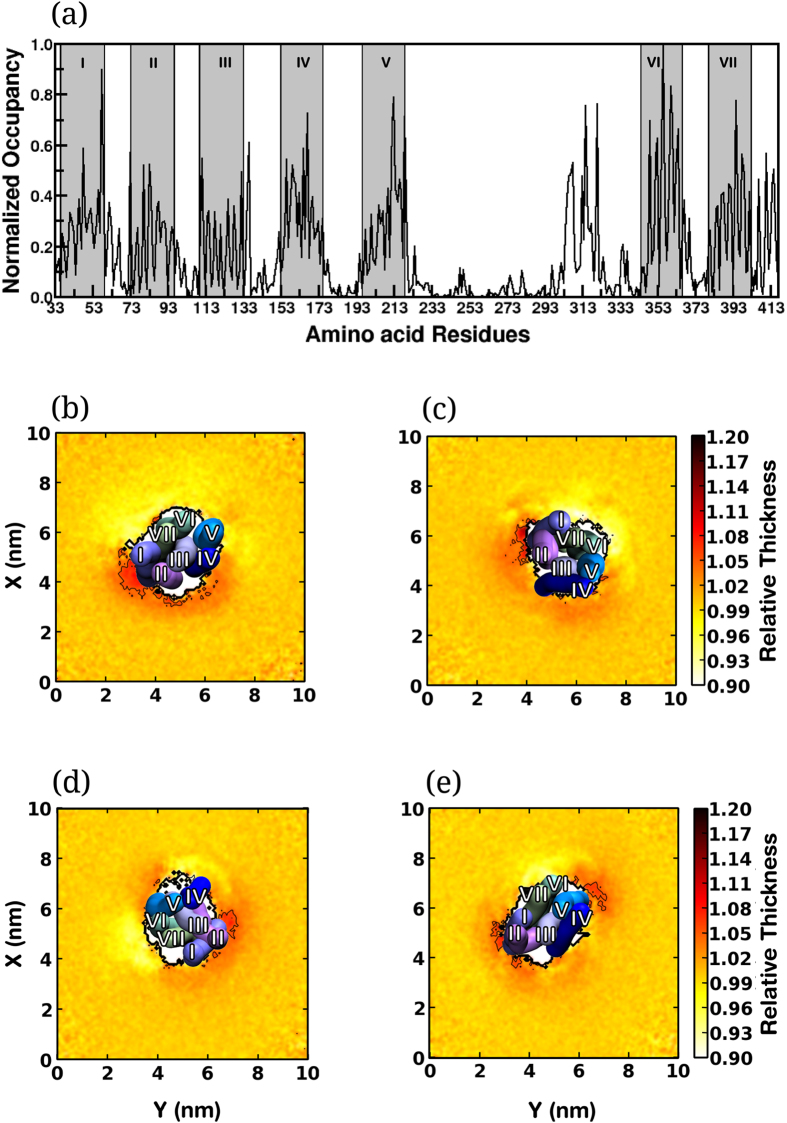
Direct and indirect cholesterol effects. (**a**) Maximum cholesterol occupancy around each residue of the receptor in the monomer regime. The values were normalized to the simulation length of the monomer regime and averaged over two monomers from all simulations in POPC/cholesterol bilayers. The gray bands depict the segments corresponding to the transmembrane helices. (**b**–**e)** Bilayer thickness profiles around the receptor in (**b**) POPC bilayers and POPC/cholesterol bilayers with (**c**) 9 (**d**) 30 and (**e**) 50% cholesterol concentration. A top view representation of the transmembrane helices of the receptors is superimposed on the plots.

The direct interaction of cholesterol with the receptor was analyzed by calculating the maximum occupancy of cholesterol at each residue of the receptor during the monomer regime of the simulations (see Methods for further details). Figure 5a shows cholesterol occupancy calculated around each residue, averaged over all simulations in POPC/cholesterol bilayers and normalized to the maximum value. The highest cholesterol occupancy was observed at transmembrane helix VI. In addition, high cholesterol occupancy was observed at transmembrane helices I and V (>0.8). Interestingly we observed comparably higher cholesterol occupancy at the third intracellular loop of the receptor (between transmembrane helices V and VI), indicating interaction of the loop with the membrane. The values calculated considering each transmembrane helix is consistent (Supplementary Fig. 3). These results suggest that the presence of cholesterol at transmembrane helix I in the monomeric regime could be related to its subsequent absence at the dimer interface (see Fig. 3), although a direct correlation is difficult.

#### Hydrophobic mismatch around transmembrane helices

Membrane cholesterol is known to regulate lipid-protein interactions by increasing the thickness of the membrane bilayer. It has been previously reported that the bilayer thickness of POPC vesicles increases from ~26 Å to ~30 Å in presence of 30% cholesterol^40^. This could give rise to ‘hydrophobic mismatch’, *i.e*., a difference in the hydrophobic lengths of transmembrane proteins and the surrounding lipid annulus, that can lead to changes in membrane protein oligomerization^41^,^42^,^43^,^44^. We analyzed the variation in the normalized bilayer thickness around the receptor monomer to directly compare the local bilayer thickness at all membrane compositions and quantitate the mismatch around the receptor. The normalized bilayer thickness profiles are shown in Fig. 5(b–e). In POPC bilayers, an increased bilayer thickness was observed at site 1 of the receptor, corresponding to transmembrane helices I and II (Fig. 5b). An increase in bilayer thickness was also observed around site 2 comprising of transmembrane helix IV, but was lower in magnitude. In POPC/cholesterol bilayers with 9% cholesterol (Fig. 5c), both sites 1 and 2 exhibit increased bilayer thickness. In POPC/cholesterol bilayers with 30 and 50% cholesterol (Fig. 5d,e), the positive hydrophobic mismatch at site 1 was reduced, and the mismatch at site 2 was increased. Interestingly, the main dimer conformations observed in our simulations (Fig. 2) correspond to the sites of the receptor in which the membrane perturbations are high. However, a direct correlation is difficult. For example, the perturbations at site 1 persist even at high cholesterol concentrations, although a homodimer at this site, comprising of transmembrane helix I was observed to be unfavorable. Cholesterol-dependent receptor dimerization therefore appears to be a complex interplay between direct and indirect effects.

### Nonannular cholesterol contributes to dimer flexibility and plasticity

**Figure 6:**
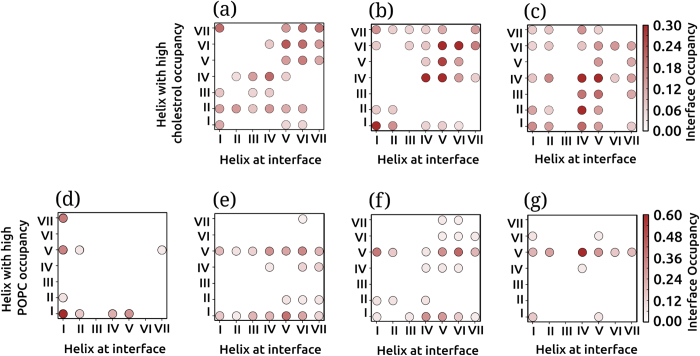
Cholesterol occupancy and its correlation to dimer interface: cholesterol as a molecular lubricant. The normalized occupancy of cholesterol (panels **a**–**c**) and POPC (**d**–**g**) at each helix in the receptor dimer plotted as a function of the helices at the dimer interface. The interface occupancy scores (see Methods) of cholesterol are shown for POPC/cholesterol bilayers with (**a**) 9 (**b**) 30 and (**c**) 50% cholesterol concentrations. The interface occupancy scores of POPC are shown for (**d**) POPC bilayers and POPC/cholesterol bilayers with (**e**) 9 (**f**) 30 and (**g**) 50% cholesterol concentrations. The interface occupancy scores were normalized to the relative probability of occurrence of the transmembrane helix at the dimer interface (obtained from Fig. 3) and highest maximum cholesterol occupancy for each receptor (from Fig. 5). The diagonal elements correspond to high occupancy of cholesterol (or POPC) at helices that constitute the dimer interface. The off-diagonal elements correspond to occupancy of cholesterol (or POPC) at the helices not at the dimer interface. The presence of cholesterol at the dimer interface is reminiscent of nonannular lipids^38^ and is believed to act as a ‘molecular lubricant’ by modulating the energetics of helix-helix interaction (see text). See Methods for further details.

Cholesterol occupancy in the dimer regime was found to be different from that in the monomer regime (see Fig. 5 and Supplementary Fig. 4). High cholesterol occupancy was observed in the dimer regime around transmembrane helices IV, V and VI, similar to the monomer regime. However, cholesterol occupancy at transmembrane helix I decreased considerably in the dimer regime. To examine if the varying cholesterol occupancies were correlated to the dimer interface, we calculated an interface occupancy score (see Methods for details). The interface occupancy for cholesterol calculated for POPC/cholesterol membrane bilayers is shown in Fig. 6(a–c). The diagonal elements in the plots correspond to high cholesterol occupancy at the transmembrane helices at the homodimer interface, and the off-diagonal elements correspond to the cholesterol occupancy away from the interface. In POPC/cholesterol bilayers with 9% cholesterol (Fig. 6a), the diagonal elements show high scores, suggesting that the cholesterol occupancy at the transmembrane helices at the dimer interface is high. The score for the off-diagonal elements, corresponding to cholesterol occupancy at receptor sites not at the dimer interface was relatively low, suggesting lower cholesterol at sites away from the dimer interface. Similarly, in POPC/cholesterol bilayers with 30% cholesterol (Fig. 6b), the scores of the diagonal elements were high, confirming the presence of cholesterol at the dimer interface. In POPC/cholesterol bilayers with 50% cholesterol (Fig. 6c), the effect was less pronounced and high cholesterol occupancy was observed at all transmembrane helices, both at the dimer interface and away from it. In sharp contrast, the occupancy of POPC was not related to the dimer interface (Fig. 6d–g). POPC occupancy was high at transmembrane helix I and shifted to transmembrane helix V with increasing cholesterol concentration, consistent with the monomer regime (Supplementary Fig. 5). Interestingly, the site of POPC association at transmembrane helix I is identical to that predicted by recent atomistic simulations^45^. *The presence of high cholesterol occupancy at the dimer interface and the lack of correlation of POPC occupancy with the dimer interface*, strongly point out the involvement of membrane cholesterol in receptor dimerization.

The high cholesterol occupancy at the dimer interface is reminiscent of nonannular sites that have been suggested to be present at inter-receptor sites as well as intra-receptor (inter-helical) sites^38^. We propose that in case of the serotonin_1A_ receptor, the nonannular sites are related to both dimer plasticity as well as the rotational flexibility of the dimer interface. From this perspective, cholesterol could be thought of acting as a ‘molecular lubricant’, and could modulate the conformational energetics of helix-helix interaction in the membrane.

## Discussion

GPCRs are important mediators of signaling networks that have been shown to be dependent on membrane cholesterol. Cholesterol appears to function in a receptor-dependent manner by modulating the structure and organization, but the molecular details of these mechanisms have been difficult to probe due to this inherent complexity. In addition, GPCR organization is important not just as an organizational principle but also as a regulatory paradigm influencing receptor cross-talk and drug efficacy. In this work, we have analyzed the dimerization of the serotonin_1A_ receptor, an important GPCR, by comprehensive coarse-grain simulations, totaling to an effective time of ~15 ms (see Methods). By analyzing the dimerization behavior of the receptor in POPC bilayers with increasing concentration of cholesterol, we are able to delineate the subtle, yet functionally relevant, effect of cholesterol on receptor association. The dimer states display four distinct conformers with sharply defined boundaries that are dependent on membrane lipid composition (Fig. 2). We propose that the dynamics of association of cholesterol molecules at receptor dimer interfaces, promotes receptor rotational flexibility and conformational plasticity, much needed for their biological activity.

An interesting aspect of the current work is that although multiple dimer interfaces are observed, they can be mapped to mainly two sites: site 1 involving transmembrane helices I and II, and site 2 comprising of transmembrane helices IV, V and VI. A continuous interplay of each of these helices and rotation of the receptor gives rise to several dimer interfaces, fine- tuned by cholesterol concentration. These effects are mediated, possibly through specific interaction. Indeed, cholesterol has been shown bind at specific sites on GPCRs by atomistic^46^,^47^,^48^,^49^ and coarse-grain simulations^24^,^36^,^37^,^50^. In addition, cholesterol association kinetics at the ns and μs time scales has been reported by NMR studies^51^. An interesting feature of our results is the observation that conformational plasticity in terms of populations of the various dimer species and rotational flexibility in terms of helices at the dimer interface are increased in presence of cholesterol (see Figs 2 and 3). Previous studies on oligomerization of related GPCRs, rhodopsin and the opioid receptor have identified similar sites on the receptor as important protein-protein contacts^52^,^53^. Molecular dynamics simulations have suggested that GPCR oligomerization is dynamic with comparable energetics of helix-helix interactions^52^,^54^. Similarly, two different dimer interfaces were observed in the oligomeric crystal structure of β_1_-adrenergic receptor^55^ and μ-opioid receptor^56^. Mutational studies combined with protein-protein docking suggested the presence of transmembrane helices IV and V in the dimer interface of the serotonin_1A_ receptor^57^. Importantly, such a dynamic association can explain the effect of cholesterol on the organization in serotonin_1A_^22^,^23^ and the neurotensin^58^ receptors. Modulation of conformational plasticity by cholesterol could contribute toward modulation of the oligomer populations. On the other hand, serotonin_2C_ receptor exhibits a high population of receptor dimers on the plasma membrane^59^,^60^, similar to the endoplasmic reticulum and Golgi, although the lipid composition differs^59^. Our work validates the dynamic nature of the receptor-receptor interface, and establishes the importance of cholesterol in regulating and modulating receptor organization.

An important feature of the current work is the identification of short-lived dimer species with unfavorable dimer interfaces. The large number of dissociations observed, especially at high cholesterol concentrations (Fig. 4c,d) points toward a comprehensive sampling of the dimerization process at the sub-ms time scale regime. Previous studies of GPCR association have focussed on oligomeric species and due to limitations in the time scales sampled, were unable to identify these unfavorable dimer interfaces^24^,^41^,^42^,^53^. The presence of the short-lived dimer species supports the importance of cholesterol in modulating the energetics of receptor-receptor interaction, thereby increasing the flexibility and plasticity of serotonin_1A_ receptor dimers.

The increased conformational plasticity of the serotonin_1A_ receptor dimer by membrane cholesterol could be relevant in cellular physiology and drug discovery. Cellular cholesterol is known to be developmentally regulated and in a cell type dependent manner^61^,^62^. This could imply that the organization of the dimers is age and cell type dependent. Given the central role of the serotonin_1A_ receptor in anxiety and depression, this would suggest an age-dependent implication in disease progression. Further, the tissue-dependent organization of GPCRs could be important in the context of drug efficacy and specificity.

In conclusion, using multiple coarse-grain simulations we have been able to identify important cholesterol-dependent organizational principles in GPCRs. The conformational plasticity of the serotonin_1A_ receptor dimer has been demonstrated to be dependent on cholesterol. By occupying nonannular sites at the dimer interface, cholesterol is suggested to modulate helix-helix interaction and directly influence the protein contacts. Our work is an important step toward understanding GPCR function in healthy and diseased conditions.

## Methods

### System setup

Multiple coarse-grain molecular dynamics simulations of two membrane embedded serotonin_1A_ receptors were performed using the MARTINI force-field^63^,^64^. Twenty simulations of 45 μs each were carried out in POPC/cholesterol bilayers with varying cholesterol concentration, corresponding to a total of 900 μs at each cholesterol concentration. The total simulation time equals 3.6 ms of coarse-grain simulation time, corresponding to 14.4 ms of effective simulation time^63^,^64^. The coarse-grain representation of the homology model of serotonin_1A_ receptor and POPC bilayers with varying cholesterol concentration (0, 9, 30 and 50%) were obtained from earlier studies^36^,^65^. Four orientations of the receptors, rotated about 90 degrees from each other were considered, with a minimum distance of 3.0 nm between the receptors. The schematic representations of the receptor and a representative initial system are shown in Supplementary Fig. 1 (also see Supplementary Table 1). Simulations of the monomeric receptor in POPC bilayers with varying cholesterol concentrations were performed under identical conditions (see Supplementary Table 2).

### Simulation parameters

All simulations and analysis were performed using GROMACS version 4.5.5 66. The systems were represented by the MARTINI coarse-grain force-field version 2.1 for the protein and version 2.0 for the lipid parameters. Non-bonded interactions were used in their shifted form with electrostatic interactions shifted to zero in the range of 0–1.2 nm and Lennard-Jones interaction shifted to zero in the range of 0.9–1.2 nm. The temperature of each molecular group in the system was weakly coupled to a thermostat at 300 K using the Berendsen thermostat algorithm with a coupling constant of 0.1 ps^67^. Pressure was maintained semi-isotropically at 1 bar independently in the plane of the bilayer and perpendicular to the bilayer using Berendsen’s barostat algorithm with a coupling constant of 0.5 ps and a compressibility of 3 × 10^−5^ bar^−1^. Initial velocities for system were chosen randomly from a Maxwell distribution at 300 K. The LINCS algorithm was used to constrain bond length. A time step of 20 fs was used for the simulations with neighbor list updated every 10 steps. Periodic boundary conditions were maintained along x, y and z direction. Simulations were rendered using the VMD software^68^ along with MARTINI secondary structure rendering scripts.

### Analysis

#### Characterization of the rotational angles sampled by the receptors

The relative orientation of the receptors was calculated from the angle between the planes defined by residues from transmembrane helices I and IV (see Supplementary Fig. 2). θ_1_ refers to the rotational angle of receptor 1 relative to receptor 2 characterized by the angle between the planes formed by the backbone beads of residues 48, 164 and 167 of receptor 1 and residue 167 of receptor 2 (residues numbered according to UNIPROT ID: P08908). Similarly, θ_2_ refers to the rotational angle of receptor 2 relative to receptor 1 characterized by the same residues. To account for reduced dimer interactions in POPC/cholesterol bilayers with 50% cholesterol concentration, an equal number of random simulations were considered for the remaining membranes.

#### Quantitative estimation of involvement of transmembrane helices at dimer interface

The transmembrane helices at the dimer interface were determined from the dimer regime by using a cut-off of 0.5 nm, using the same methodology as in our earlier work^36^. Long-lived dimer species were identified as those where no subsequent dissociation (larger than cut-off) was observed. Transient dimers where characterized as those in which a dissociation event (minimum distance of 1.5 nm) was observed subsequently in the trajectory.

#### Maximum cholesterol occupancy around each residue of receptor

Maximum cholesterol occupancy is defined as the maximum (normalized) time for which a cholesterol molecule remains associated with a particular site. The definition was based on our earlier work^24^,^36^,^43^.

#### Hydrophobic mismatch around transmembrane helices

The normalized bilayer thickness profile around the monomer was calculated from the phosphate bead distances of the bilayer based on previous work^43^. A value >1 indicates local thickening (positive hydrophobic mismatch) and a value <1 indicates local thinning (negative hydrophobic mismatch).

#### Cholesterol occupancy at dimer interfaces

To quantify the cholesterol occupancy at the dimer interface, we calculated normalized lipid occupancy for each dimer interface which we term as ‘interface occupancy score’. The ‘interface occupancy score’ is defined for each helix in a given dimer conformation as the product of the normalized cholesterol occupancy and the probability of that dimer conformation. In the interface occupancy matrix, the diagonal elements correspond to high cholesterol occupancy at the helices at the dimer interface and the off-diagonal elements correspond to high cholesterol occupancy at transmembrane helices not occurring at the dimer interface. To reduce noise in the calculations, only the helices with at least 70% probability of occurrence at the interface were considered. Similarly, only helices with a maximum cholesterol occupancy of 0.7 were considered.

## Additional Information

**How to cite this article**: Prasanna, X. *et al*. Cholesterol-dependent Conformational Plasticity in GPCR Dimers. *Sci. Rep.* **6**, 31858; doi: 10.1038/srep31858 (2016).

## Supplementary Material

Supplementary Information
