# Supplementary material for: Cholesterol-dependent Conformational Plasticity in GPCR Dimers
Source: Sci Rep. 2016 Aug 18;6:31858. doi: 10.1038/srep31858 (PMC4989139; doi:10.1038/srep31858)
Supplement: Supplementary Information [file srep31858-s1.pdf]

SUPPORTING INFORMATION

Cholesterol-dependent Conformational Plasticity in GPCR Dimers

Xavier Prasanna,[†] Durba Sengupta[†]* and Amitabha Chattopadhyay[‡]*

[†]CSIR-National Chemical Laboratory, Dr. Homi Bhabha Road, Pune 411 008, India

[‡]CSIR-Centre for Cellular and Molecular Biology, Uppal Road, Hyderabad 500 007, India

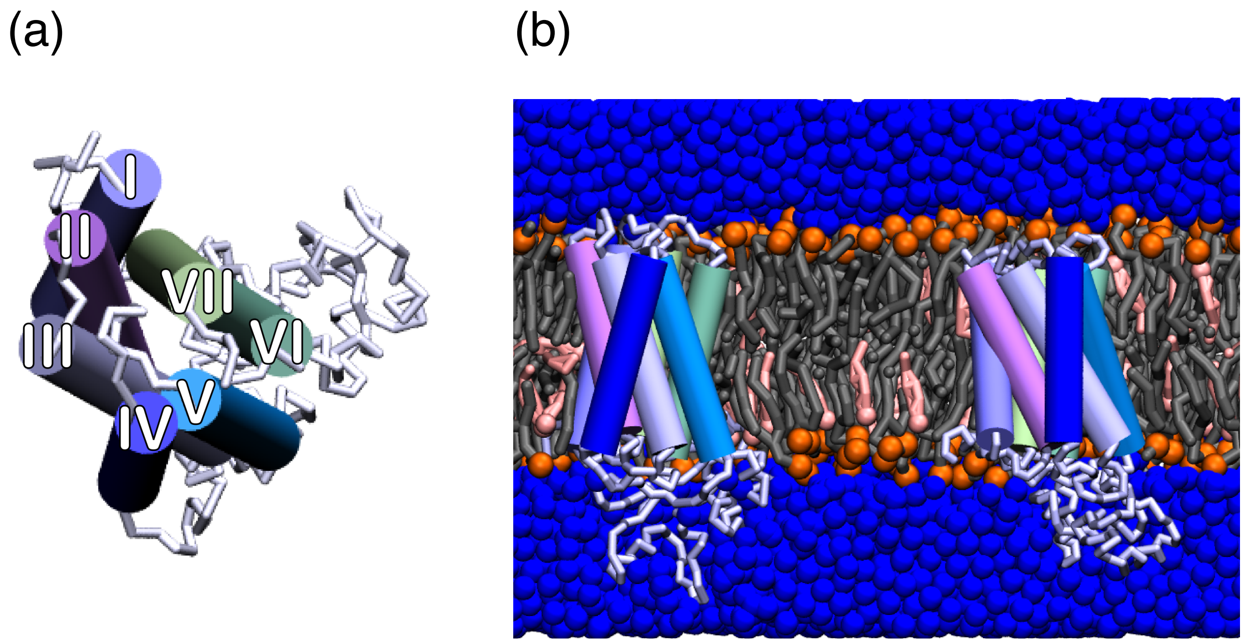

Supplementary Figure 1. A schematic representation of the serotonin$_{1A}$ receptor: (a) Top view of the receptor with individual helices numbered; (b) starting structure of the two monomers in POPC bilayers with 30% cholesterol. The receptors are shown in same colors as that in panel (a). Phospholipid molecules are shown in gray, the phosphate beads of the phospholipids are shown in orange, cholesterol is shown in pink and surrounding water molecules are shown in blue.

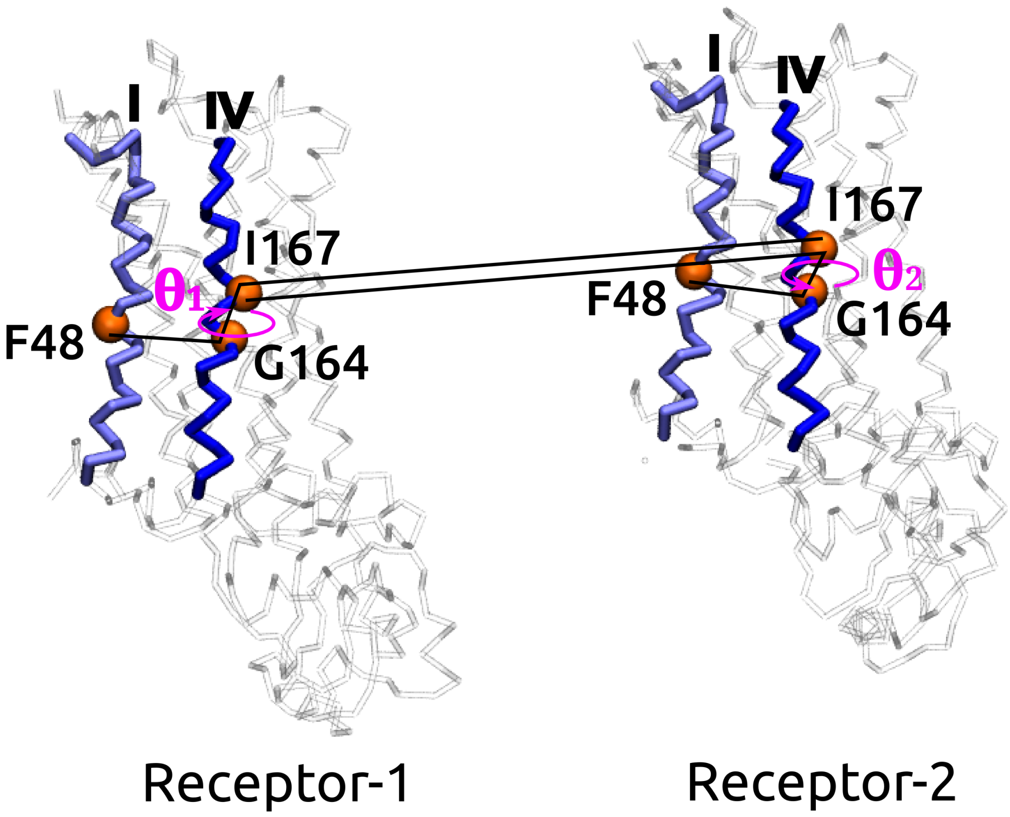

Supplementary Figure 2. Angles θ_1 and θ_2 representing the relative orientations in the dimeric state: A schematic representation of the relative orientations of the two receptors. θ_1 refers to the relative orientation of receptor 1 relative to receptor 2 and θ_2 is the relative orientation of receptor 2 relative to receptor 1. The relative orientation is defined as the angle between the planes formed by the backbone beads of residues 48, 164, 167 of receptor 1 and residue 167 of receptor 2, as shown in the figure. The helices containing these residues, *i.e.*, transmembrane helices I and IV, are shown.

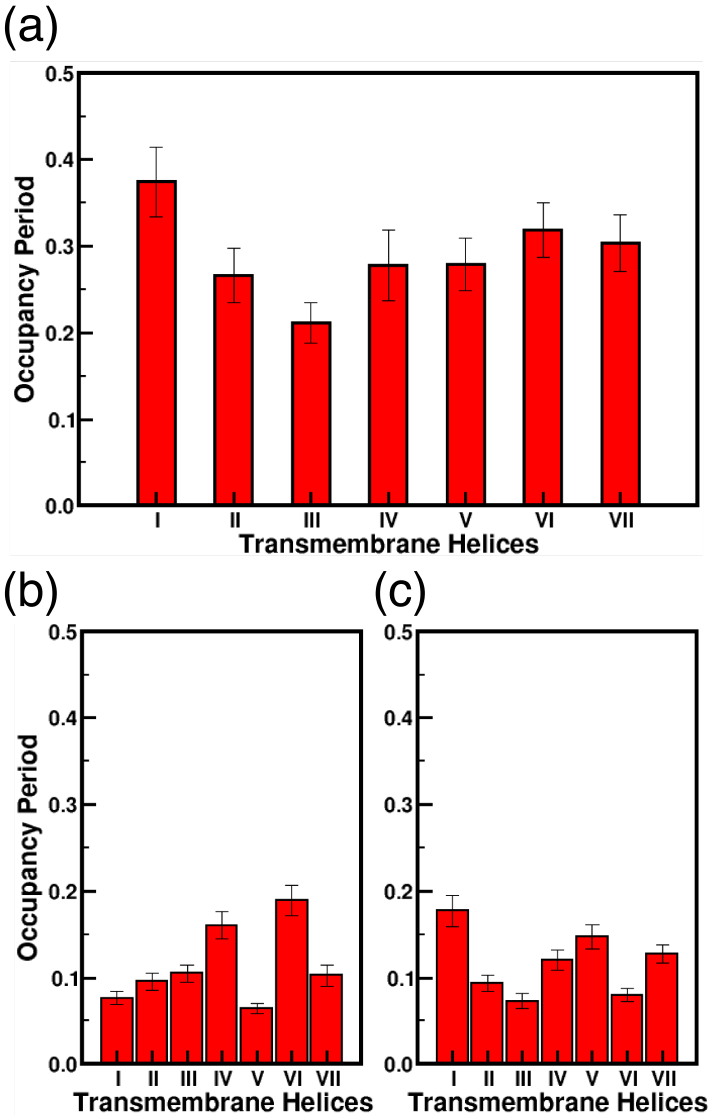

Supplementary Figure 3. **Maximum cholesterol occupancy around each transmembrane helix in the monomer regime.** The values are reported for (a) the transmembrane helix as a whole and (b) upper and (c) lower leaflets separately. The values have been normalized to the simulation length of the monomer regime and averaged over the two receptors from all simulations in POPC/cholesterol bilayers.

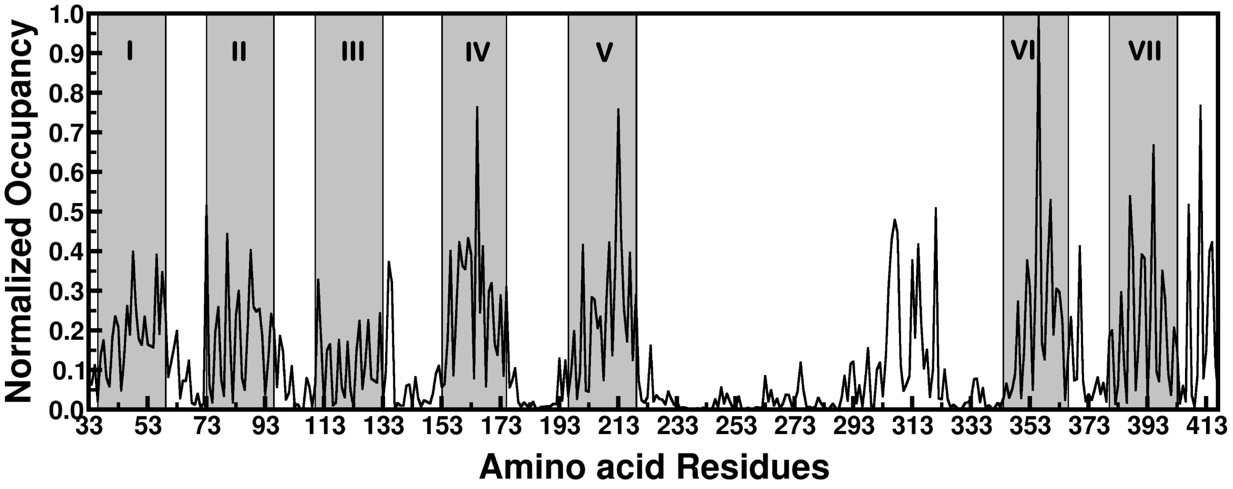

Supplementary Figure 4. **Maximum cholesterol occupancy around each residue of the receptor in the dimer regime**: The values have been normalized to the simulation length of the dimer regime and averaged over the two receptors from all simulations in POPC/ cholesterol bilayers. The gray bands depict the segments corresponding to the transmembrane helices.

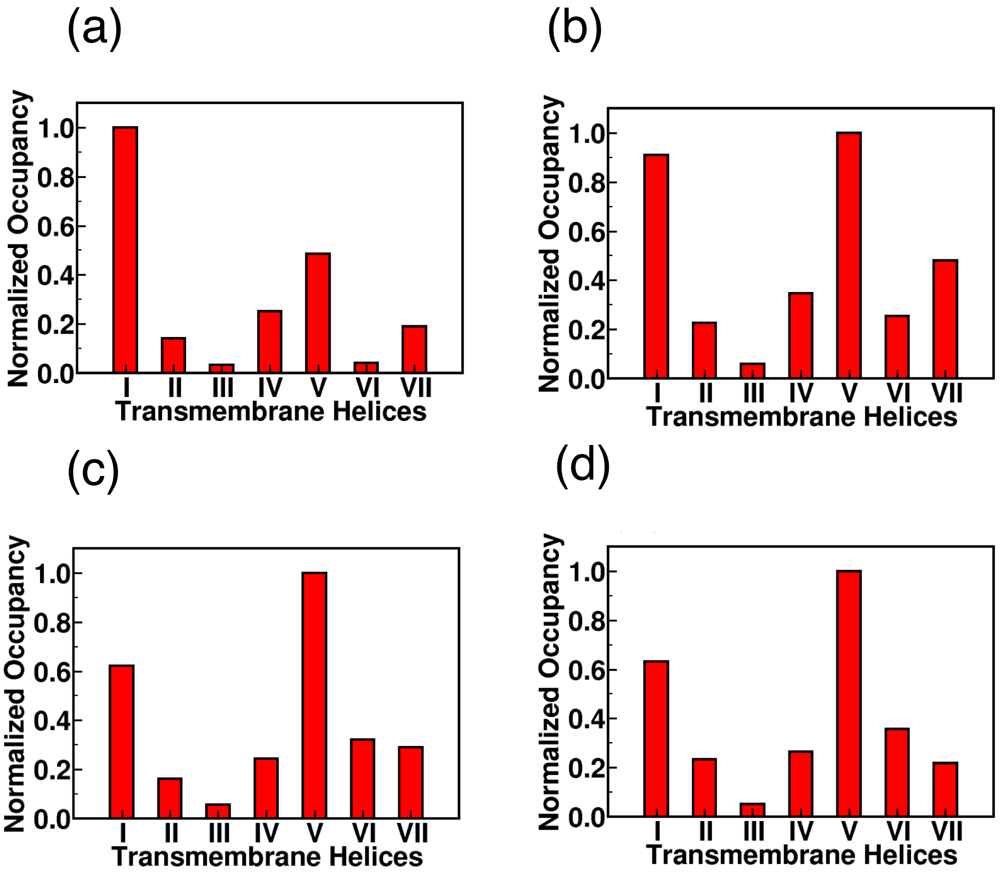

Supplementary Figure 5. Maximum POPC occupancy around the transmembrane helices of the receptor in the monomer regime: The maximum occupancy was calculated from the phosphate bead of the phospholipid molecule for simulations in (a) POPC bilayers and POPC/cholesterol bilayers with (b) 9 (c) 30 and (d) 50% cholesterol concentrations. The values have been normalized to the simulation length and averaged over the two receptors in the monomer regime.

Supplementary Table 1

Summary of simulations performed

System	Initial Minimum Distance (nm)	Simulation Number	Simulation Length (μs)	Number of Molecules Receptor	Number of Molecules POPC	Number of Molecules Cholesterol
Dimer simulations						
POPC	3.2	20	45	2	568	0
POPC/ 9% cholesterol	3.5	20	45	2	520	48
POPC/ 30% cholesterol	3.6	20	45	2	520	158
POPC/ 50% cholesterol	3.1	20	45	2	520	260
Monomer simulations						
POPC	-	1	25	1	284	0
POPC/ 9% cholesterol	-	1	25	1	260	24
POPC/ 30% cholesterol	-	1	25	1	260	78
POPC/ 50% cholesterol	-	1	25	1	260	130

Supplementary Table 2
Quantitative estimation of number of transient associations

System	Number of transient associations
POPC	8
POPC/ 9% cholesterol	28
POPC/ 30% cholesterol	40
POPC/ 50% cholesterol	48
